# Safety and efficacy of platelet-rich plasma injection for treatment of erectile dysfunction: a prospective randomized controlled study

**DOI:** 10.1186/s12610-024-00232-3

**Published:** 2024-10-08

**Authors:** Ahmed M. Ragheb, Amr M. Lotfy, Mohamed Fahmy, Akrm A. Elmarakbi

**Affiliations:** Department of Urology, Faculty of Medicine, Beni‑Suef University, Beni‑Suef, Egypt

**Keywords:** PRP, PRFM, Erectile dysfunction, Plasma riche en Plaquettes (PRP), Matrice de Fibrine riche en Plaquettes (MFRP), Dysfonction érectile

## Abstract

**Background:**

Affected sexual relationships affect both the quality of life of men and women. Platelet-derived therapies are becoming increasingly popular in various medical and surgical fields because of their ability to aid in tissue healing and vascular remodeling. This study aimed to assess the safety and effectiveness of platelet-rich plasma (PRP) injections in treating patients with erectile dysfunction (ED).

**Results:**

Fifty-two participants with mild to moderate ED were divided into two groups: group A, who received three PRP penile injections, and group B, who received three saline injections (5 ml for each injection site). The International Index of Erectile Function (IIEF) was used to evaluate all participants. A month after the last injection, the PRP group's IIEF was 16.12 ± 1.25, while the placebo group's was 15.99 ± 1.21 (*p* = 0.683). Following a 3-month period, the IIEF for the PRP group was 16.44 ± 1.17, while the placebo group's was 16.31 ± 1.06 (*p* value = 0.653). Following a 6-month period, the IIEF for the PRP group was 16.35 ± 1.45, while that for the placebo group's was 16.23 ± 1.19 (*p* = 0.727). In terms of IIEF, there was no significant difference between the two groups after one, three, and six months of treatment.

**Conclusion:**

In brief, our research revealed no data to support the application of PRP injections in the management of mild-to-moderate ED.

## Résumé

**Contexte:**

Une altération des relations sexuelles affecte à la fois la qualité de vie des hommes et des femmes. Les thérapies dérivées des plaquettes deviennent de plus en plus populaires dans divers domaines médicaux et chirurgicaux, en raison de leur capacité à aider à la guérison des tissus et au remodelage vasculaire. La présente étude visait à évaluer l’innocuité et l’efficacité d’injections de plasma riche en plaquettes (PRP) dans le traitement des patients atteints de dysfonction érectile (DE).

**Résultats:**

Fifty-two participants atteints de dysfonction érectile légère à modérée ont été divisés en deux groupes: le groupe A, qui a reçu trois injections de PRP dans le pénis, et le groupe B, qui a reçu trois injections de solution saline (5 ml pour chaque site d’injection). L’indice international de la fonction érectile (IIEF) a été utilisé pour évaluer tous les participants. Un mois après la dernière injection, l’IIEF du groupe PRP était de 16,12 ± 1,25, tandis que celui du groupe placebo était de 15,99 ± 1,21 (*p* = 0,68). Trois mois après la dernière injection, l’IIEF pour le groupe PRP était de 16,44 ± 1,17, tandis que celui du groupe placebo était de 16,31 ± 1,06 (valeur *p* = 0,65). Enfin, 6 mois après la dernière injection, l’IIEF pour le groupe PRP était de 16,35 ± 1,45, tandis que celui du groupe placebo était de 16,23 ± 1,19 (*p* = 0,73). En ce qui concerne l’IIEF, il n’y avait pas de différence significative entre les deux groupes après un, trois et six mois de traitement.

**Conclusion:**

En bref, notre recherche n’a révélé aucune donnée en faveur de l’application des injections de PRP dans la prise en charge de la dysfonction érectile légère à modérée.

## Background

The inability to obtain or sustain a hard penile erection that is strong enough for sexual activity is known as erectile dysfunction. The process of achieving erection is multidimensional and complex, and is caused by the interaction of various physiological systems. Immediately after sexual stimulation, neural- and endocrine-mediated venous dilation causes blood to flow to fill the corpus cavernosum lacunae. Venous outflow is restricted in response to engorgement, maintaining erection until detumescence. As previously stated, the production and maintenance of an erection may be hampered by complications in any of the hormonal neurological, psychological or mechanical components of this process [1].

According to a Goldstein et al. study, the prevalence of ED appears to vary from 37.2% in Brazil to 48.6% in Italy [2]. The prevalence of ED is much higher in aging populations—more than 50% of cases occur in men between the ages of 40 and 70 [3, 4]. An ED-specific quality of life questionnaire can be used to evaluate the psychosocial consequences since it has a significant impact on patients' quality of life [5].

Common comorbidities that affect ED include obesity, hypertension, and diabetes mellitus (DM) [6]. Large-scale population studies worldwide have also revealed high rates of psychological discomfort and depression, which aggravate the physical constraints of ED [7, 8].

Intracavernosal injections (ICI) and penile implants are examples of more invasive treatment options for ED, although less invasive behavioral methods and medication such as PDE5 inhibitors have also been employed. In recent years, new and controversial therapies have emerged that are labeled as "regenerating," including platelet-rich plasma (PRP) injections, intracavernous stem cell therapy (SCT), and low intensity extracorporeal shock wave therapy (Li-ESWT) [9].

A number of medical and surgical specialties have reported an increase in the use of platelet-derived therapies [10]. PRP is regarded as a restorative and regenerative therapy that attempts to treat the underlying mechanical causes of ED rather than just its symptoms, due to their high concentration of growth factors and cytokines (e.g., TGF-β, IGF-1, and VEGF), which are well known to have healing effects on muscle and soft tissue, these therapies are thought to stimulate the endogenous regenerative potential of human tissue to return to normal structure and function [11].

Red and white blood cells are extracted from whole blood by centrifuging it through a separator gel to produce PRP. There is a more than four-fold increase in platelets and other plasma proteins in the resultant supernatant; this concentrate is then administered via injection. In order to address the issue of early washout with PRP, more recent approaches to extending the wound-healing and anti-inflammatory properties of platelets have been focused on making a fibrin matrix (platelet rich fibrin matrix, or PRFM) to bind the platelets and prevent extravasation from the injection site [12, 13].

In the current research, we aimed to assess the safety and effectiveness of PRP injections compared to placebo in patients who had been complaining of mild to moderate ED for at least six months through a prospective, randomized, double-blind, placebo-controlled study.

## Patients and methods

### Study design and setting

A prospective, double-blinded, randomized, placebo-controlled study, that was performed in the outpatient clinic of the Andrology unit of the Urology department, Faculty of Medicine, Beni-Suef University Hospital between April 2022 and April 2023.

### The characteristics of participants

#### Inclusion criteria

Patients aged 20–80 years, presented with mild-to-moderate ED (pre-intervention ED scoring IIEF5 = 12–21) for at least 6 months despite a stable heterosexual relationship. Patients were asked to suspend all ED therapy, engage in sexual activity at least once per week, and record the results in a Sexual Encounter Profile (SEP) diary for the length of the research.

#### Exclusion criteria

Patients with a history of previous major pelvic surgery that may have affected erectile function, prior history of penile fracture or priapism, previous pelvic radiation, abnormal morning serum testosterone level defined as a value lower than 300 ng/dL ± 5%, or greater than 1197 ng/dL ± 5%, those presented with psychogenic ED or Peyronei disease, and who had received hormonal treatment for prostate cancer in the past or present were all excluded and eliminated from the study.

### Intervention

#### Pre-intervention evaluation

A detailed history was obtained from all participants regarding their age, occupation, location of residence, and any special habits such as drug or alcohol consumption and smoking, the frequency and quality of morning erections, as well as the sexual response to any physical or visual stimuli, age of the wife, and compliance with sex. Medical history, particularly conditions that might affect sexual function, such as DM, hypertension, and coronary heart disease. Surgical history, history of accidents, and genital injuries were also recorded. All participants were examined for 2ry sexual characteristics, gynecomastia, body weight, and the existence of surgical scars. Genital examination focusing on testicular size and consistency, spermatic cord examination, urethral meatus for size and location, and penis for tenderness or plaques. The IIEF score [14] was used to evaluate all patients. Blood samples were obtained from each participant, including a complete blood count (CBC), morning serum testosterone, coagulation profile, Hgb A1C, and lipid profile.

#### PRP preparation and administration

A venipuncture was performed at the clinic. Ten milliliters of whole blood were placed into two different collection tubes. The samples were centrifuged at 6,000 RPMs. PRP was then mixed in a 1:10 ratio with a 10% calcium chloride solution to convert fibrinogen to fibrin. Typically, this procedure produces 5 mL of injectable PRFM (activated PRP) in each tube. PRFM was selected to prevent early washout and to improve the retention of local products.

An FDA-approved autologous platelet separator (Magellan Autologous Platelet Separator; Arteriocyte Medical Systems, Hopkinton, MA) was used to process patient samples randomly assigned to receive PRP. Specifically, PRP was delivered into a separate sterile syringe after being automatically separated from the anti-coagulated whole blood within approximately 15 min. Administration was performed within 10 min of the final preparation. Patients were placed in the supine position. A topical anesthetic cream containing 25 mg lidocaine and 25 mg prilocaine (Eutectic Mixture of Local Anesthetics (AstraZeneca, UK) was applied at the injection site. A penile tourniquet is clipped around the base of the penis. In the mid-penile region, there is a 1 cm variation in the injection locations. To reduce platelet damage, 5 mL of PRFM was pumped into each corpus cavernosum over the course of 2 min, carefully retracting the needle to improve PRFM distribution within the erectile tissue. The entire procedure was performed under sterile conditions. After administration, the penis was further compressed and the penile shaft was wrapped in a dressing. Following surgery, patients were monitored in the clinic for 20–30 min to look for any possible side effects or complications. The penile tourniquet was removed 20 min after the injections, and the patients were released. All patients were instructed to remove the compression bandage at home 4 h after the injection.

Patients of both PRP and placebo groups had received 3 sessions of PRP and normal saline penile injection respectively with 15 ± 3 days treatment interval; 10 ml had been injected at each session.

#### Post-injection follow up

The patients were assessed at 1, 3, and 6 months following injection using the IIEF score, which has been authenticated and translated into Arabic [15]. Via phone calls and inquiries about safety, possible negative experiences for which no medical care was sought were assessed.

### Statistical analysis

#### Sample size

Software created by a computer was used to perform randomization. The sample size was calculated at a power of 90%, effect size of 0.2, and number of measures of the IIEF-5 score of three times. We ran repeated measures ANOVA with the interaction of timing factor and grouping factor on the IIEF-5 score and two groups of cases (PRP and placebo groups). The acquired sample size was 52 patients (26 in each arm). The sample size was calculated using Windows G-power 3.0.

#### Statistical method

With the use of SPSS (statistical package for social science) version 25 (Armonk, NY: IBM Corp.), the data was tabulated and analyzed. Depending on the type of data, mean ± SD was used to represent quantitative data, while numbers and percentages were used to represent qualitative data. The comparison and relationship between two qualitative variables were examined using the chi-square test (χ2), and the comparison between two groups that included quantitative variables with a normal distribution was examined using the Student’s t-test. Statistical significance was set at *p* < 0.05.

#### Descriptive statistics

Quantitative information is displayed as mean ± SD, but qualitative data are shown as numbers and percentages based on the type of data.

#### Analytic statistics

The chi-squared test and Student’s t-test were used to examine the correlation and comparability of qualitative parameters, with a *P*-value of less than 0.05 for statistical significance.

## Results

This prospective study included 52 patients who were divided into two groups. PRP group (26 patients injected with PRP) and placebo group (26 patients injected with normal saline).

According to Table 2, the average age of PRP group was 52.2 ± 4.33 years, while placebo group's mean age was 52.5 ± 5 years (*p* value = 0.818). In PRP group, the mean ED duration was 12.87 ± 4.04 months, while in placebo group, it was 11.8 ± 3.6 months (*p* = 0.318). In terms of medical comorbidities, the PRP group had 10 cases (38.4%), 7 cases (26.9%), and 4 cases (15.3%) of DM, hypertension, and dyslipidemia, respectively, while 7 cases (26.9%), 4 cases (15.3%), and 2 cases (7.6%) in the placebo group had the same comorbidities.

There were no statistically significant differences between the two groups with regard to age, ED duration, or comorbidities. Also we can observe that placebo group's mean HbA1C was 5.38 ± 0.66 and that of PRP group was 5.6 ± 0.79 (*p* value = 0.281). Patients of PRP group had a mean IIEF of 15.97 ± 1.38, while placebo group's patients had a mean IIEF of 15.73 ± 1.72 (*p* = 0.582). Again, there was no statistically significant difference between the two groups with regards to HbA1C and IIEF (Pre).

Table 3, showed that, following a month, PRP patients' IIEF was 16.12 ± 1.25, while placebo group patients' IIEF was 15.99 ± 1.21 (*p* value = 0.683). After three months, PRP group patients' IIEF was 16.44 ± 1.17 and placebo group patients' IIEF was 16.31 ± 1.06 (*p* value = 0.653). After six months, PRP group patients' IIEF was 16.35 ± 1.45 while placebo group patients' IIEF was 16.23 ± 1.19 (*p* value = 0.727). So, we can observe that, there was an improvement in both PRP and placebo group's IIEF at 1, 3 and 6 month time points compared to pre-injection status but the difference was not statistically significant. Also, there was no significant difference between both PRP and placebo groups, suggesting that PRP is not superior to placebo.

As shown in Table 1, among the participants who presented to the follow-up evaluations, 4/26 (15.4%) patients achieved an improvement in the PRP group compared to 7/26 (26.9%) in the placebo group (*p* < 0.308) at 1 month. At this time point, 11.5% (95% CI: 10.4 to 11.6) less patients treated with PRP injections developed an improvement in IIEF score compared to placebo. At 3 months, 9/26 (34.6%) patients achieved an improvement in the IIEF score after PRP injections versus 15/26 (57.7%) after placebo (*P* = 0.095) so, at this time point we can observe that 23.1% (95% CI: 11.85 to -15) less patients treated with PRP injections developed an improvement in the IIEF score compared to placebo. At six months, 11/26 (42.3%) patients reported an improvement in the IIEF score with PRP injections versus 10/26 (38.5%) with placebo (*P* < 0.777), and the risk difference between the two groups was 3.8% (95% CI: 13.8 to 13.2).

**Table 1:** Comparative data of the 2 groups about number and percentage of patients achieving improvement in the IIEF score at the follow up evaluations

Patients with improvement in IIEF score	PRP group	Placebo group	RD (95% CI)	*P* value
1 Month	4/26 (15.4%)	7/26 (26.9%)	-11.5% (10.4,-11.6)	0.308
3 Months	9/26 (34.6%)	15/26 (57.7%)	-23.1% (11.85, -15)	0.095
6 Months	11/26 (42.3%)	10/26 (38.5%)	3.8% (13.8 to -13.2)	0.777

a. Data presented as numbers and percentages

b. Statistical test used: chi-square test

c. *Abbreviations*: *PRP* Platelet rich plasma, *IIEF* International index of erectile function, *CI* Confidence interval, *RD* Risk difference

### Safety evaluation

During the injection and follow-up periods, neither group experienced any transient hemorrhagic adverse events (hematuria, local petechial bleeding, or ecchymosis) or other adverse effects.

## Discussion

The persistent or repeated inability to achieve and/or sustain a penile erection strong enough for satisfying sexual activity, including fulfilling sexual performance, is known as ED [16].

PRP is a promising biotechnology that has been demonstrated to promote and accelerate healing of soft tissues and bones [17]. It works on cells to induce vascular expansion (angiogenesis) and multiplication (mitogenesis), both of which enhance healing. Additionally, they are loaded with cytokines and growth factors (GFs) that influence angiogenesis, inflammation, and cell division. [18].

The current study found that the mean age of the PRP group of patients was 52.2 ± 4.33 years, whereas the placebo group's mean age was 52.5 ± 5 years (*p* value = 0.818). Additionally, the mean ED duration in months was 12.87 ± 4.04 in the PRP group and 11.8 ± 3.6 in the placebo group (*p* value = 0.318) as shown in Table 2. This is comparable to the results of a recent study by Wong et al. [19], in which the patients' mean age was 54.93 years (± 8.31 years), but their mean ED duration was more than 24 months.

**Table 2:** Baseline characteristics of the study population

	PRP group (*N* = 26)	Placebo group (*N* = 26)	*P*. Value
Age (Years)	52.2 ± 4.33	52.5 ± 5	0.818
Duration of ED (months)	12.87 ± 4.04	11.8 ± 3.6	0.318
HbA1C	5.6 ± 0.79	5.38 ± 0.66	0.281
IIEF (Pre)	15.97 ± 1.38	15.73 ± 1.72	0.582
Co-morbidities			
DM	10 (38.46%)	7 (26.92%)	0.375
HTN	7 (26.92%)	4 (15.38%)	0.308
Dyslipidemia	4 (15.38%)	2 (7.69%)	0.385

a. Data presented as number and percentages or mean ± SD as appropriate

b. *P* values determined by the Chi-square test (χ2) & the Student t-test

c. *Abbreviations*: *ED* Erectile dysfunction, *IIEF* International index of erectile function, *DM* Diabetes mellitus, *HTN* Hypertension

In this work, there was no significant difference between both groups regarding IIEF after 1, 3 and 6 months of treatment as show in Table 3. This is in line with Masterson et al. [20] in the United States in 2023; there was no difference between the PRP and placebo groups, despite the investigators founded a significant minimal clinically important difference (MCID) between them. This suggests that PRP is not better than a placebo. Banno et al. published a comparable study on a small group of male patients in their facility who received PRP injection (one injection only) in addition to their ED's standard of care, which included medication and vacuum therapy [21], where PRP effects were evaluated at least four weeks after injection. Only 9 patients were included in the study Table 4, the sample size was insufficient to detect an effect, and the score difference did not reach statistical significance. This was in conflict with a study by Taş et al. that was released in 2021 [22] in Turkey, where 35 men with ED were injected with 3 mL of PRP into the corpora cavernosa. Although 61.29% of subjects showed improvement in erectile function (*P* < 0.001), yet there was no discernible rise in orgasmic function or sexual desire. Also there was no placebo group for this study.

**Table 3:** Comparative data of the 2 groups about IIEF score evaluation during the follow up periods

IIEF	PRP group (*N* = 26)	Placebo group (*N* = 26)	*P*. Value
Initial evaluation	15.97 ± 1.38	15.73 ± 1.72	0.5642
At 1 month	16.12 ± 1.25	15.99 ± 1.21	0.6838
At 3 months	16.44 ± 1.17	16.31 ± 1.06	0.6537
At 6 months	16.35 ± 1.45	16.23 ± 1.19	0.7273
Evaluation of both groups through time	0.538	0.321	***P*. value**

a. Data presented as mean ± SD

b. *P* values determined by the Student t-test

c. *Abbreviation*: *IIEF* International index of erectile function

**Table 4:** Previous similar studies investigating PRP in ED patients compared to the current research

Study	Design	Number of patients	PRP preparation	Number of injections	Safety	Efficacy
**Chalyj et al. (2015**) [25]	prospective cohort study	75 patients, 30 patients a PRP,30 a PRP with PDE5 I, 15 PRP	(1) PRP activated with 10% calcium chloride solution (30 patients received this ICI); (2) PRP activated with 10% calcium chloride combined with PDE5i medication (30 in this group); (3) Inactivated PRP (15 patients received this IC injection)	2 injections 4 weeks apart	No adverse events	A statistically significant rise in IIEF5 score (*P* > 0.046), PSV (*P* = 0.005), and RI (*P* = 0.001) was noted in group 1. PSV (*P* = 0.028), RI (*P* = 0.129), and the IIEF5 score (*P* = 0.046) all showed improvements in group 2. A statistically significant difference was observed in the IIEF5 score (*P* < 0.05), PSV, and RI (*P* > 0.05) values in group 3
**Banno et al. (2017**) [21]	prospective cohort study	9 patients	Could not be found	1 injection in combination with PDE5 I or vacuum	No adverse events	Average IIEF5 score prior to injection was 15.6 (range 12–20) and at least 4 weeks post injection the score was 19.9 (range 11–27). The difference in scores did not achieve statistical significance
**Matz et al. (2018**) [26]	Retrospective	5 patients	Venipuncture was performed in the clinic. Two separate collection tubes were filled with 9 mL of whole blood. The samples were centrifuged at 6,000 RPMs for six minutes, and the supernatant was separated from the remaining blood sample using a proprietary system. Ten percent calcium chloride solution was then added to the PRP in a 1:10 ratio, converting fibrinogen to fibrin	1 injection	Mild pain	Potential efficacy is suggested, but larger sample and control are required
**Alkhayal et al. (2019) ** [27]	Prospective study	61 patients in PRP group	Automatic dual spin Magellan Arteriocyte machine	1 injection of prp	No adverse events	Mean follow-up was for 11 weeks only. Mean IIEF5 score before treatment was 12.5 (range 5–20) (considered mild-to-moderate ED) and post treatment mean IIEF5 score was 17 (range 5–24) (mild ED), *P* < 0.001. Although this study does show a positive result with the use of PRP, there is no placebo-controlled group in the study
**Taş et al. (2021) ** [22]	Prospective study	35 patients	Whole blood: 8-min centrifuge at 2800 rpm, followed by plasma: 10-min centrifuge at 3500 rpm (1000–2000 × 10^3^/μL); 3 m	3 injections with 2 weeks apart	No adverse events	IIEF5 score improved from 18 to 20 (*P* < 0.001), however despite this improvement the score remained within the mild IIEF5 ED
**Poulios et al. (2021) ** [23]	Randomized, double blind, placebo-controlled study	60 patients, 30 in PRP and 30 in placebo group	Magellan Autologous Platelet Separator (NR); 5 mL/corpora	2 injections 4 weeks apart	No adverse events	20/29 (69%) of men in the PRP group vs. 7/26 (27%) of the men in the placebo group achieved MCID, *p* < 0.001
**Shaher et al. (2023) ** [24]	Prospective randomized placebo controlled	109 patients (9 excluded) to PRP or saline placebo	Whole blood: 5-min centrifuge at 2500 rpm, followed by plasma: 10-min centrifuge at 3500 rpm (NR); 3 mL/corpora via 1 mL at 3 sites	3 injections with 2 weeks apart	No adverse events	Significant improvement at 1,3 months follows up that slightly dropped at 6- month .76% patient 0f PRP group had an improved IIEF with MCID compared to only 18% in the saline group
**Masterson et al. (2023) ** [20]	Prospective, randomized, double-blind, placebo controlled, clinical study	61 patients	Arthrex Angel PRP system (NR); 2.5 mL/corpora	2 injections 4 weeks apart	1 minor adverse event in each group	There was no difference between groups in percentage of men meeting minimum clinically important difference at 1 month: 14 (58.3%) in platelet-rich plasma vs 15 (53.6%) in placebo (*P* = .730). Mean International Index of Erectile Function–Erectile Function domain changed from 17.4 (95% CI 15.8–19.0) to 21 (17.9–24.0) at 1 month in men receiving platelet-rich plasma, vs 18.6 (17.3–19.8) to 21.6 (19.1–24.1) in the placebo group; however, there was no significant difference between groups (*P* = .756)
**Current study**	Prospective randomized placebo controlled	52patients, 26 in PRP & 26 in saline group	Magellan Autologous Platelet Separator; 5 ml/corpora	3 injections with 15 ± 3 days treatment interval	No adverse events	Prior to injection, the average IIEF5 score for the PRP group was 15.9, whereas the saline group's score was 15.7. At 1, 3 and 6 months, the PRP group outperformed the saline group, although the difference in scores did not reach statistical significance

*Abbreviations*: *RI* Resistance index, *PSV* Peak systolic velocity, *PRP* Platelet rich plasma, *IIEF* International index of erectile function, *MCID* Minimum clinically important difference, *PDE5I* Phosphodiesterase type 5 inhibitor, *ED* Erectile dysfunction, *RPMs* Revolutions per minute, *ICI* Intracorporal injection, *CI* Confidence interval

Poulios et al. conducted the first double blind, randomized, placebo-controlled trial of PRP for ED in Greece that same year [23]. Sixty men with mild, mild to moderate or moderate ED were randomized 1:1 to receive ICI PRP or a saline placebo treatment in the form of two injections with four weeks apart. They observed a statistically significant increase in IIEF and SEP scores at every follow-up time point.

Another study from Egypt was reported in 2023 by Shaher et al. [24]. 109 participants in the trial were randomized to receive either a saline placebo or PRP. Three mL injections were given to each patient, separated by two weeks. SEP and IIEF scores were obtained at one, three, and six month follow up time points. By the end of the first and third months, there was a noticeable improvement that somewhat declined at the end of the sixth month Table 4.

During the injection and follow-up period in both groups, no additional side effects or temporary hemorrhagic adverse events (hematuria, local petechial hemorrhage, or ecchymosis) were reported in this investigation. This was consistent with the results of Banno et al. [21] and Poulios et al. [23] who reported no significant treatment-related side effects. In addition to induration at the first injection site, Wong et al. [19] observed that there was no infection, hematoma, or pain at the injection site.

The limitations of this study include a small sample size and the short duration of follow-up (6 months), which may be inadequate to assess long-term differences. Additionally, the protocol we utilized was based on previous PRP studies, and it is possible that our protocol of 3 injections with 15 ± 3 days treatment interval will not achieve optimal results. It is possible that a different interval between injections may lead to greater changes in IIEF score. More research into patient selection, protocol optimization, mean platelet volume and the number of platelets in the solution is needed.

## Conclusions

In brief, our research offered no evidence that could support the efficacy of PRP injections in the management of patients suffering from mild to moderate ED. More research is required to determine the safety and effectiveness of PRP injections for ED in large-scale population studies with extended follow-up times.

## Data Availability

No datasets were generated or analysed during the current study.

## Abbreviations

DM: Diabetes mellitus
ED: Erectile Dysfunction
GFs: Growth factors
ICI: Intra-cavernosal injections
IIEF-5: The International Index of Erectile Function Questionnaire
Li-ESWT: Low intensity extracorporeal shock wave therapy
MCID: Minimal clinically relevant difference
PRFM: Platelet rich fibrin matrix
PRP: Platelet-Rich Plasma
RPMs: Revolutions per minute
SCT: Stem cell therapy
SEP: Sexual Encounter Profile
